# The Influence of Gamma Radiation on Different Gelatin Nanofibers and Gelatins

**DOI:** 10.3390/gels10040226

**Published:** 2024-03-26

**Authors:** Carmen Gaidau, Maria Râpă, Gabriela Ionita, Ioana Rodica Stanculescu, Traian Zaharescu, Rodica-Roxana Constantinescu, Andrada Lazea-Stoyanova, Maria Stanca

**Affiliations:** 1 Research and Development National Institute for Textiles and Leather-Division Leather and Footwear Research Institute, 93 Ion Minulescu Street, 031215 Bucharest, Romania; carmen.gaidau@icpi.ro (C.G.); rodica.constantinescu@icpi.ro (R.-R.C.); 2 Faculty of Materials Science and Engineering, POLITEHNICA Bucharest National University of Science and Technology, 313 Splaiul Independentei, 060042 Bucharest, Romania; maria.rapa@upb.ro; 3 “Ilie Murgulescu” Institute of Physical Chemistry, 202 Splaiul Independentei, 060021 Bucharest, Romania; ige@icf.ro; 4 Department of Analytical and Physical Chemistry, University of Bucharest, 4-12 Regina Elisabeta Bd., 030018 Bucharest, Romania; istanculescu@nipne.ro; 5 Horia Hulubei National Institute of Research and Development for Physics and Nuclear Engineering, 30 Reactorului Str., 077125 Magurele, Romania; 6 National Institute for R&D in Electrical Engineering ICPE-CA, 313 Splaiul Unirii, P.O. Box 149, 030138 Bucharest, Romania; traian.zaharescu@icpe-ca.ro; 7 National Institute for Laser, Plasma and Radiation Physics, 409 Atomistilor Street, 077125 Magurele, Romania; andrada@infim.ro

**Keywords:** gelatin nanofibers, gelatins, gamma irradiation, structural characteristics

## Abstract

Gelatin nanofibers are known as wound-healing biomaterials due to their high biocompatible, biodegradable, and non-antigenic properties compared to synthetic-polymer-fabricated nanofibers. The influence of gamma radiation doses on the structure of gelatin nanofiber dressings compared to gelatin of their origin is little known, although it is very important for the production of stable bioactive products. Different-origin gelatins were extracted from bovine and donkey hides, rabbit skins, and fish scales and used for fabrication of nanofibers through electrospinning of gelatin solutions in acetic acid. Nanofibers with sizes ranging from 73.50 nm to 230.46 nm were successfully prepared, thus showing the potential of different-origin gelatin by-products valorization as a lower-cost alternative to native collagen. The gelatin nanofibers together with their origin gelatins were treated with 10, 20, and 25 kGy gamma radiation doses and investigated for their structural stability through chemiluminescence and FTIR spectroscopy. Chemiluminescence analysis showed a stable behavior of gelatin nanofibers and gelatins up to 200 °C and increased chemiluminescent emission intensities for nanofibers treated with gamma radiation, at temperatures above 200 °C, compared to irradiated gelatins and non-irradiated nanofibers and gelatins. The electron paramagnetic (EPR) signals of DMPO adduct allowed for the identification of long-life HO^●^ radicals only for bovine and donkey gelatin nanofibers treated with a 20 kGy gamma radiation dose. Microbial contamination with aerobic microorganisms, yeasts, filamentous fungi, *Staphylococcus aureus*, *Escherichia coli*, and *Candida albicans* of gelatin nanofibers treated with 10 kGy gamma radiation was under the limits required for pharmaceutical and topic formulations. Minor shifts of FTIR bands were observed at irradiation, indicating the preservation of secondary structure and stable properties of different-origin gelatin nanofibers.

## 1. Introduction

The development of new wound care biomaterials is relevant for the high incidence of skin injuries and hard-to-heal wounds with morbidity and mortality risks [1]. The polyamphiphilic properties of gelatin enable its structuring in versatile materials with advanced functionalities for food [2,3,4,5], cosmetic [6,7,8,9,10], pharmacy [11,12,13], and medical applications [14,15,16,17].

Commercial gelatin [18] is processed from porcine skin (46% of total production), bovine hides (29.4%), bovine bones (23.1%), or fish (1.5%), and it represents a universal additive in the food industry as an emulsifier, stabilizer, and foaming or encapsulating agent [5]. The most advanced materials processed using gelatin are polyelectrolyte complexes [19], nanoemulsions [20,21], nanoliposomes [22], nanogels [23], and nanofibers [17,24].

Gelatin is one of the most available biomaterials compared to native collagen due to its higher solubility and lower cost.

The greater polar structures and the easy availability of tripeptides, which are known for cell-binding affinity, make gelatin a more bioactive material compared to native collagen [25]. The use of different-origin biomasses for designing new added-value biomaterials represents the topic of many reported research studies [26,27,28,29]. In the same line, our research shows the potential of processing different-origin gelatins into nanofibers with high contact surface area and using non-toxic solvents.

Nanofibers are ideal structures for wound healing stimulation because of their high surface area to volume ratio and porous morphology that allow cell attachment and proliferation [30]. The potential of including active substances through electrospinning can provide antimicrobial and anti-inflammatory properties that contribute to healing mechanisms [31,32,33,34,35].

The efficiency of gelatin-based nanofibers for wound healing stimulation was previously evaluated through in vitro and in vivo tests and correlated with gelatin structural properties, which can be preserved through electrospinning [34,36].

The sterilization of wound healing patches through gamma radiation represents an important step before commercialization. The influence of the gamma radiation dose on the microbial load and the structure of gelatin-based nanofibers represents an essential topic of knowledge not covered by the literature. Our research aimed to investigate the influence of gamma irradiation doses on gelatin-based nanofibers prepared using different-origin, collagen-based by-products compared to their gelatins of origin.

It was reported that the collagen membranes fabricated by casting blends of gelatin with 0.7% lemongrass essential oils can be preserved against oxidation induced by gamma irradiation radicals compared to membranes without essential oils [37]. Also, the influence of riboflavin on different-origin gelatins subjected to gamma irradiation showed the retention of free radicals on oxygen and modification of the secondary structure of the protein. Another paper showed that the denaturation temperature values and the amide structure analyses of higher molecular proteins (native collagen and gelatins) exposed to gamma irradiation suggested a protective shield effect compared to lower molecular proteins of keratin [38]. Similarly, the structured collagen of tanned leathers showed slight modifications in physical–mechanical and physical–chemical characteristics after exposure to a 25 kGy irradiation dose [39].

Many studies on the influence of gamma irradiation on collagen or the extracellular matrix (ECM) have been devoted to in vivo tissue behavior under medical treatment with relatively low radiation doses of 7–12 kGy [40,41]. The influence of gamma irradiation between 2 and 30 kGy on AlloDerm acellular tissue matrix showed that even if the crosslinking occurs at low gamma doses and molecular fragmentation occurs at higher gamma doses, the in vitro fibroblast cell proliferation was not affected [41]. The methacrylated gelatin-based hydrogels with applications for bone-substituting material fabrication proved to have stable behavior after sterilization with a 30 kGy gamma radiation dose compared to methacrylated hyaluron with altered dissolution behavior. Both sterilized hydrogels induced the proliferation of human-bone-marrow-derived mesenchymal stem cells, opening a promising path for hybrid hydrogel applications [42].

Some gamma irradiation experiments on different collagen materials showed that high energy exposure, atmospheric conditions, hydration state, and preparation methods are the main factors that can highly influence their structures. The protein scissor occurs in a dry state, while intermolecular crosslinking can occur in the hydrated state of materials subjected to gamma radiation doses between 50 and 100 kGy [43].

Several pharmaceutical materials were intensively studied for radiolysis process identification concerning their potential efficiency alteration [44,45]. The influence of their compositions and the susceptibility to oxidative degradation, breaking, or crosslinking under the influence of reactive radicals generated under gamma irradiation requires structural and functional investigations of every kind of material due to their unpredictable behavior.

It is recognized that knowledge regarding the behavior of proteins and peptides under the action of gamma radiation is currently limited [46]; therefore, the present paper wishes to contribute to the understanding of these phenomena.

For this purpose, the properties of gelatins and nanofibers made from them were compared in their initial state and after gamma irradiation with doses of 10, 20, and 25 kGy. Thus, the behavior of analyzed materials through chemiluminescence (CL) emission was investigated at various temperatures, as well as the presence of free radicals induced and retained by gelatin or gelatin nanofibers after irradiation (EPR), the changes in the secondary structure and functional groups (assessed through Attenuated total reflectance—Fourier transform infrared spectroscopy, ATR-FTIR) and also the microbiological load depending on the dose of irradiation.

According to our knowledge, no investigation has been reported earlier in the literature that highlights the behavior of gelatins of various origins compared to the nanofibers made from them and further subjected to gamma irradiation. The research showed that a dose of 10 kGy ensures compliance with the requirements for pharmaceutical products; furthermore, this dose is considered harmless for food products [44]. The hypothesis that increasing the surface area to volume ratio and porous morphology (scanning electron microscopy or SEM analysis) of gelatin nanofibers allows the enhanced adhesion of bacteria and fungi on their surfaces compared to the gelatins of their origin was confirmed by our research. The chemiluminescence emission of non-irradiated and irradiated gelatin nanofibers and gelatins presented similar intensities, including up to 200 °C and higher values for nanofibers compared to gelatins at higher temperatures up to 250 °C, revealing the oxidative potential processes. EPS spectra allowed for the identification of HO^●^ radicals only on nanofibers treated with a dose of 20 kGy due to the predominance of recombination processes against bond scissions, as confirmed through FTIR.

## 2. Results and Discussion

### 2.1. Gelatin Characterization

**Figure 1:**
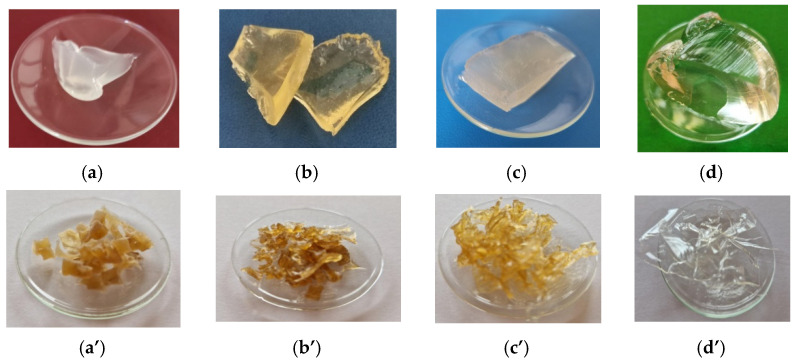
Gelatins extracted from: (**a**,**a’**) bovine hide; (**b**,**b’**) donkey hide; (**c**,**c’**) rabbit skin; (**d**,**d’**) fish scales.

It is already known that the bovine native collagen is a suitable biomaterial for replacing the extracellular matrix (EMC), thus ensuring high biocompatibility and being favorable for cell adhesion and proliferation in the wound healing process [30,47]. The collagen nanofibers prepared using gelatins of various origins (Figure 1) allow for exploiting a high variety of collagens, with new potential for wound healing and facile methods of fabrication [48].

The main characteristics of gelatins extracted from different hides, skins, and fish scales with spinnable properties are presented in Table 1.

The extracted gelatins showed a relatively large range of characteristics with significant variations in the viscosity and strength of the gelatin, which, however, did not prevent the fabrication of nanostructures with a large contact surface and, therefore, with superior efficiency to two-dimensional structures.

### 2.2. Scanning Electron Microscopy (SEM) Analysis

**Figure 2:**
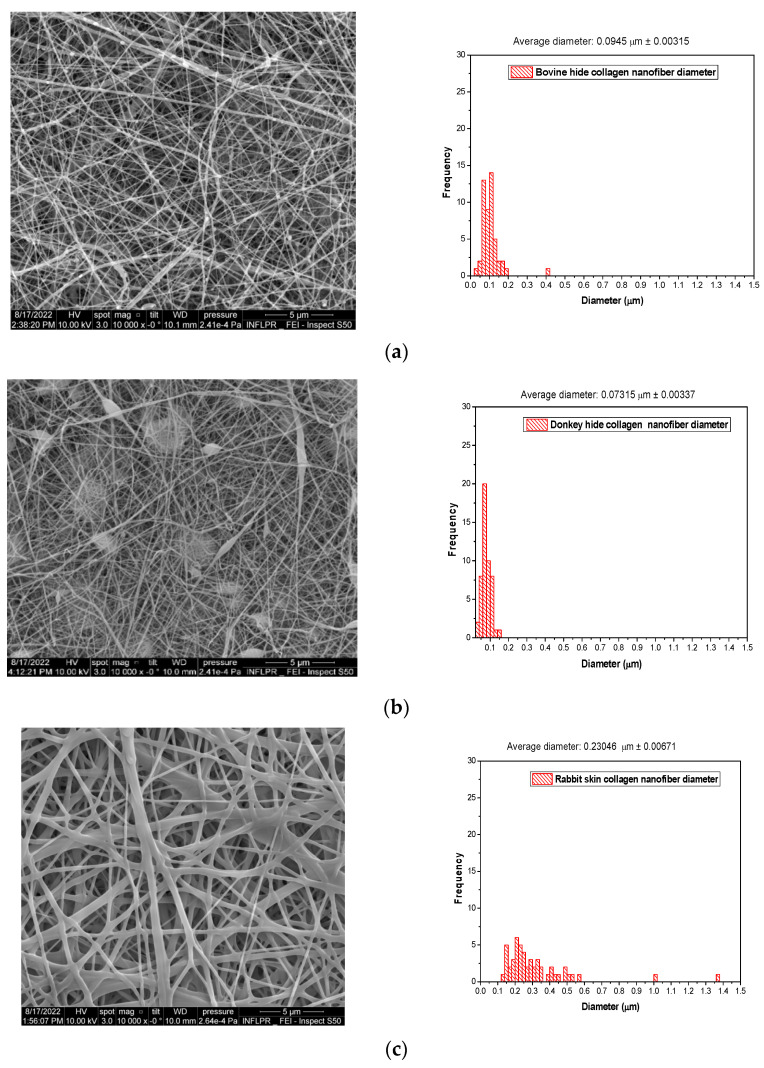

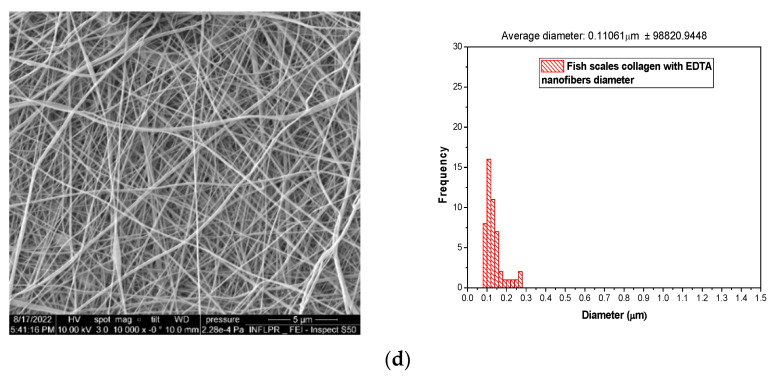
SEM images of gelatin nanofibers from gelatins of different origins: (**a**) bovine gelatin nanofibers; (**b**) donkey gelatin nanofibers; (**c**) rabbit gelatin nanofibers; (**d**) fish scale gelatin nanofibers.

Different-origin gelatins behave specifically in response to spinning and generate nanofibers with diameter sizes of 94.50 nm in the case of bovine origin collagen, 73.50 nm for donkey collagen, 230.46 nm for rabbit collagen nanofibers, and 110.60 nm for fish scale collagen nanofibers. These results show a variety of nanofiber morphologies for different gelatin origins. The studied rabbit skin gelatin and fish scale gelatin generate the most uniform nanofibers (Figure 2c,d). Branched nanofibers were fabricated from rabbit skin gelatin. It is known that rabbit skin and fish bowl gelatins are the most adhesive, which is attributed to the collagen composition and the high content of molecule subunits in gelatin [49]. The presence of the RGD (arginine–glycine–aspartic acid) sequence of amino acids identified in fish scale collagen [50] allows cell adhesion to be stimulated and skin repair to be improved. The lower concentrations of proline and hydroxyproline and higher concentrations of serine, threonine, and methionine of fish skin collagen compared to bovine collagen were also reported [51]. Extensive studies on electrospinning conditions (viscosity-crosslinking, flow rate, voltage) for pork gelatin nanofiber fabrication revealed that the average diameter is between 112 and 211.1 nm and the viscosity, voltage, and flow rate are factors influencing the average diameter [25]. In our research, we showed that the gelatin of origin is the most important factor in the fabrication of uniform gelatin nanofibers. The gelatin nanofiber average diameter size decreased in the following order: rabbit > fish > bovine > donkey.

### 2.3. The Efficacy of Antimicrobial Preservation

The aim of this research was to correlate the gamma irradiation doses for the production of new wound healing biomaterials based on gelatin nanofibers with their properties to identify the optimum conditions for their preservation. This topic is of high interest for functionalized implants with bioactive materials that are subjected to a sterilization process that most often can affect the conformation of the proteins and therefore their bioactivity [42].

The evaluation of the antimicrobial preservation of different gelatin nanofibers and the gelatins from which they were prepared with three gamma irradiation doses can provide valuable and original information on the influence of nanostructure on the preservation of their bioactive properties.

Table 2 shows that the total number of aerobic microorganisms (TAMC) and the total number of yeasts and filamentous fungi (TYMC) are higher on nanofibers prepared using bovine hide gelatin, donkey hide gelatin, and fish scale gelatin in comparison with the gelatins attributable to the higher surface area to volume ratio. The tested Gram-positive and Gram-negative strains and yeast were identified on untreated nanofibers, but they were absent after the treatment with the 10 kGy gamma irradiation dose. The concentrations of TAMC and TYMC on treated nanofibers with 10 kGy gamma irradiation dose were under 100 CFU/g, the admissible limit for pharmaceutical products [52], and this can be accepted as the lowest dose for biomaterials based on collagen nanofiber preservation. Further studies will follow the protocols for statistical determinations of sterilization conditions under gamma irradiation [53]. It is noteworthy that the gelatins do not have a microbial load, probably due to the natural ability of collagen to resist infections [29]. The antioxidant properties of rabbit gelatin and bovine collagen hydrolysate were measured and reported in previous publications [33,54] and can explain the antimicrobial properties.

### 2.4. Chemiluminescence Spectroscopy

**Figure 3:**
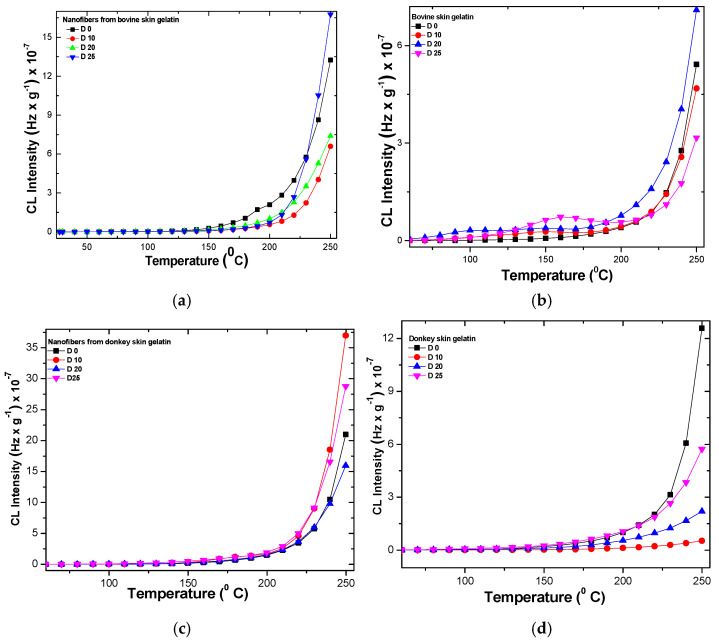

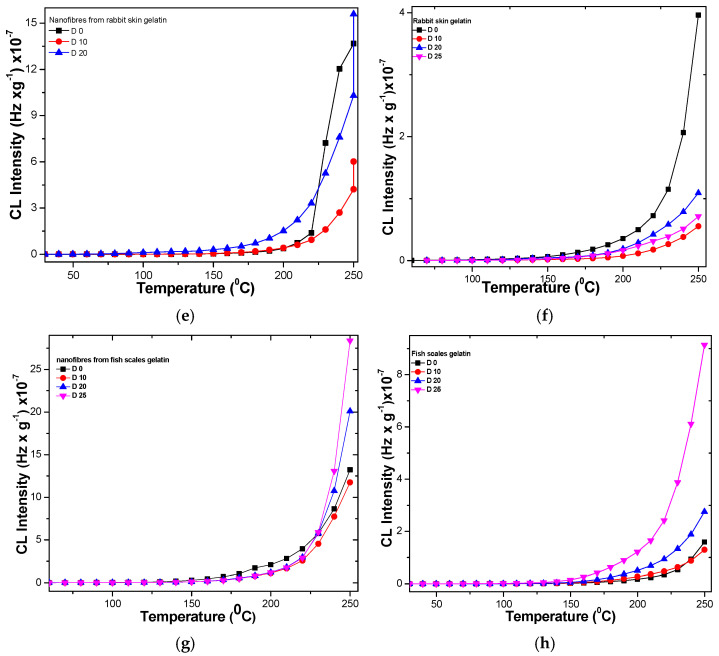
Chemiluminescent behavior of gelatin nanofibers (**a**,**c**,**e**,**g**) and gelatins (**b**,**d**,**f**,**h**) depending on temperature.

The behaviors of different gelatin nanofibers and gelatins under temperatures up to 250 °C investigated through chemiluminescence emission are presented in Figure 3. The chemiluminescence emission of nanofibers is two or even three times higher than the chemiluminescence of gelatins, as expected. The chemiluminescence of rabbit skin gelatin nanofibers at 250 °C could not be recorded. The chemiluminescence intensity of irradiated gelatin nanofibers seems to be higher at 25 kGy doses (20 kGy for rabbit skin gelatin nanofibers) at 250 °C compared to non-irradiated nanofibers and gelatins (Figure S1). The bovine gelatin irradiated with a dose of 20 kGy has shown the most intense chemiluminescent signal compared to non-irradiated gelatin and gelatins irradiated with 10 kGy and 25 kGy doses. Donkey gelatin nanofibers irradiated with 10 kGy and 25 kGy doses presented the highest CL intensity signal compared to the non-irradiated gelatin nanofibers and donkey gelatin (Figure 2d and Figure S1). Rabbit gelatin nanofibers had stable behavior compared to non-irradiated gelatin nanofibers and gelatin up to 250 °C. Rabbit gelatin nanofibers irradiated with doses of 20 kGy showed higher chemiluminescent intensity compared to non-irradiated gelatin nanofibers. Fish scale gelatin nanofibers irradiated with 20 kGy and 25 kGy doses recorded the highest chemiluminescent signals compared to non-irradiated samples starting at 240 °C, while the fish scale gelatin showed a higher chemiluminescent signal at the same gamma irradiation doses starting at 200 °C (Figure S1). The chemiluminescent intensities of fish scales and donkey gelatin nanofibers were the highest compared to all other gelatin nanofibers. The chemiluminescent signals for irradiated gelatin nanofibers started to have higher intensities compared to non-irradiated gelatin nanofibers at higher temperatures compared to gelatins irradiated at the same doses.

The increase in chemiluminescence intensity is an indication of carbonyl and hydroxyl groups’ accumulation in polymers due to the degradation effect generated by fillers and additives with effects on polymer structure [55], which, in our case, was due to the gamma irradiation. The thermal resistance of polymers is also influenced by the energy accumulated in their structure, which, in our case, was due to the different compositions in amino acid and the more ordered nanostructure in the case of collagen nanofibers. The presence of oxidable components or contaminants can also influence their stability to degradation under gamma irradiation. The least stable are the non-irradiated donkey and rabbit gelatin samples. The stability is common up to temperatures of 200 °C, after which increasing temperature accelerates oxidation. If for the control nanofibers the starting temperature of oxidation is 150 °C, for the nanofibers irradiated with a dose of 10 kGy it is 220 °C, at 20 kGy it is 203 °C, and it is 208 °C for the dose of 25 kGy. There is a competition between oxidation and recombination. The behavior of nanofibers irradiated with a dose of 25 kGy at a temperature higher than 225 °C is similar to that of non-irradiated gelatin nanofibers, which means that the more stable phase refragments (Figure S1).

### 2.5. EPR Spectroscopy

The electron paramagnetic resonance (EPR) spectra of DMPO adduct, a sensitive trapper for HO^●^ radical, were recorded in the case of gelatin and gelatin nanofibers in the initial and irradiated states. The EPR spectra detected the presence of HO^●^ radicals only for some gelatin nanofibers exposed to a 20 kGy gamma irradiation dose.

**Figure 4:**
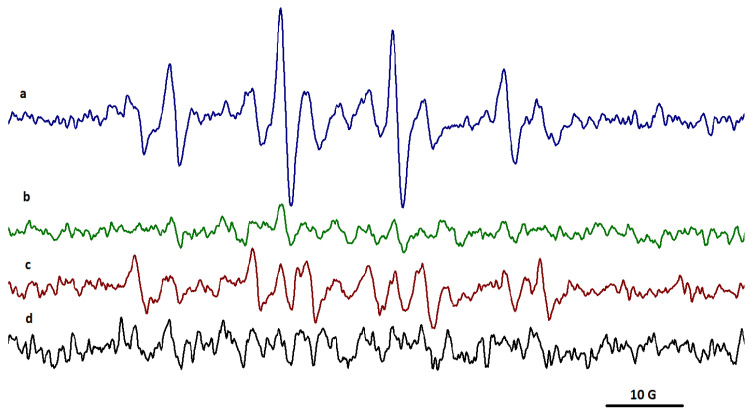
The EPR spectra of DMPO adducts in (**a**) bovine hide gelatin nanofibers, (**b**) rabbit skin gelatin nanofibers, (**c**) donkey hide gelatin nanofibers, and (**d**) fish scale gelatin nanofibers after exposure to a 20 kGy irradiation dose.

The EPR spectrum for DMPO adducts in nanofibers obtained from rabbit skin gelatin indicates the presence of HO^●^ radicals with two components: aN = 14.95 G and aH = 14.55 G (Figure 4a and Figure S2a).

The spectra highlighted the presence of HO^●^ radicals for nanofibers obtained from bovine hide gelatin and donkey hide gelatin (Figure 4a,c and Figure S2a). The EPR spectra of DMPO adduct from bovine hide gelatin nanofibers showed two components, radicals centered on carbon with the parameters aN = 14.90 G and aH = 14.74 G and on oxygen (HO^●^) with the parameters aN = 15 G and aH = 14.50 G.

In the case of donkey hide gelatin nanofibers, the two components were the radicals centered on carbon with the parameters aN = 15.60 G and aH = 22.65 G (86.20%) and on oxygen (HO^●^) with the parameters 14.95 G and aH = 14.80 G (13.80%).

**Figure 5:**
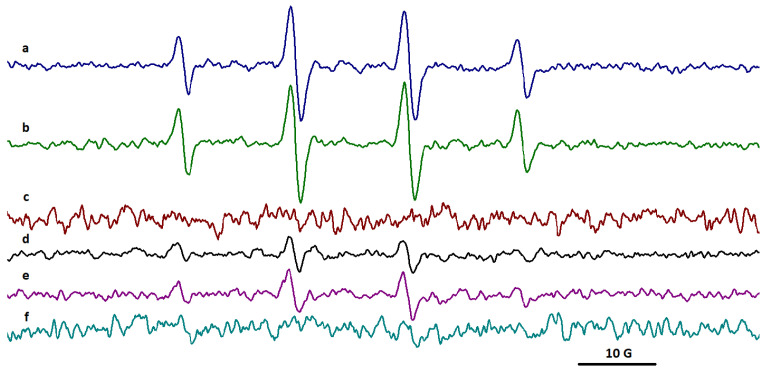
EPR spectra for DMPO adducts in (**a**,**b**) bovine, (**c**) rabbit, (**d**,**f**) donkey, and (**e**) fish scale gelatin nanofibers after 30 min. Initial signals (**a**,**d**) and signals after 30 min (**b**,**c**,**e**,**f**).

The EPR spectra of DMPO adduct from gelatin nanofibers recorded after 30 min revealed the presence of HO^●^ radicals only for bovine and donkey hide gelatin nanofibers (Figure 5b,f and Figure S2b). No adduct signal was observed for rabbit or fish skin gelatin nanofibers. In the case of bovine and donkey hide gelatin nanofibers, the EPR signal of HO^●^ adduct increased over time.

The spectra recorded over time highlighted the presence of HO^●^ radicals only in the case of gelatin nanofibers in correlation with the higher chemiluminescence signals measured for gelatin nanofibers at temperatures above 200 °C. The EPR signal of nanofibers obtained from bovine gelatin was higher (aN = 14.90 G and aH = 14.74 G) compared to the smaller signal of nanofibers obtained from donkey gelatin (aN = 14.95 G and aH = 14.69 G).

### 2.6. ATR-FTIR

**Figure 6:**
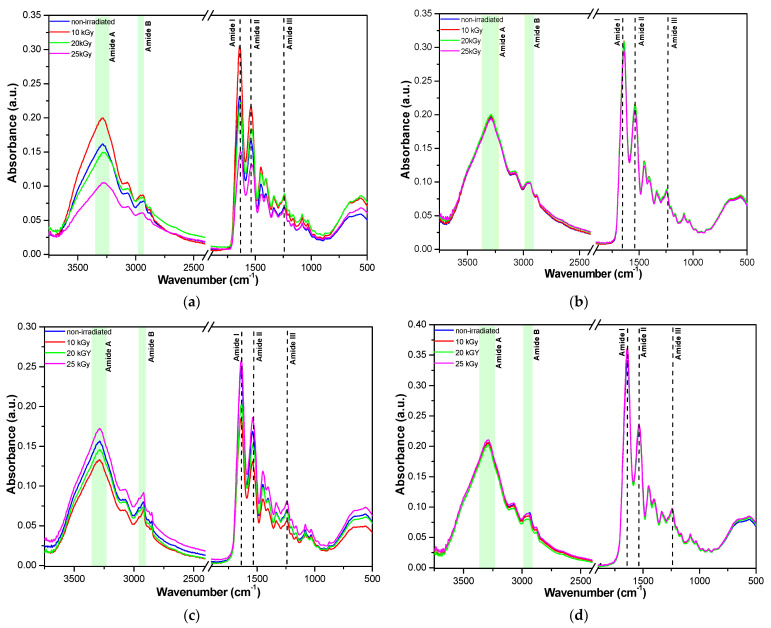

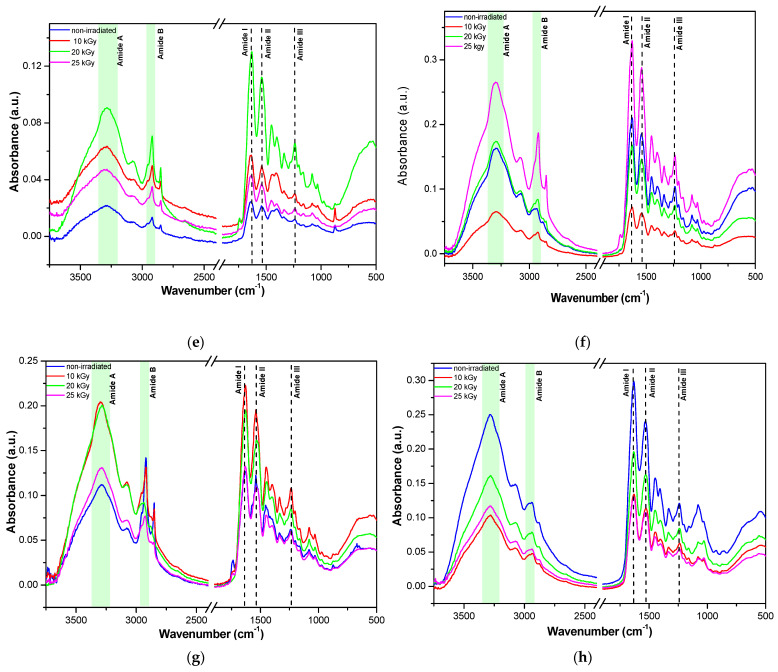
FTIR spectra of bovine (**a**), donkey (**b**), rabbit (**c**), and fish (**d**) gelatin nanofibers and bovine (**e**), donkey (**f**), rabbit (**g**), and fish (**h**) gelatins before (blue line) and after gamma irradiation at 10 kGy (red line), 20 kGy (green line), and 25 kGy (magenta line).

FTIR spectroscopy may offer a plethora of data on the sample composition, crystallinity/amorphicity, protein secondary structure, weak van der Waals, and hydrogen bond interactions, as well as stability, by analyzing the vibration bands’ position, shape, and intensity before and after various physical chemical changes, such as gamma irradiation, thermal denaturation, etc. [56,57,58,59]. The FTIR spectra of bovine, donkey, rabbit, and fish nanofibers and gelatins before and after gamma irradiation are shown in Figure 6. The FTIR spectra of gelatins compared with the nanofiber spectra showed irregular variations in intensity and shape due to the inherent difficulty of sample analysis as a consequence of intrinsic solid material hardness and local collagen structures.

Amide A, amide B, amide I, amide II, and amide III bands characteristic of collagen- based materials are given in Table 3, being in good agreement with previous data [37,38,39].

The shifts to lower wavenumbers observed in Table 3 correlate with participation in hydrogen bonds [56]. Band position modifications are also due to different amino acid compositions and secondary structures, as expected for collagen of terrestrial and marine organism origin. Thus, hydroxyproline content and/or hydroxylation degree of proline amino acids are lower in marine collagens, and the collagen from codfish showed a higher electrophoretic mobility due to lower molecular weights [56]. Also, intramolecular ß cross-linking dimers were lower than Υ cross-linking trimers in collagen obtained from the skin of codfish when compared with mammalian collagens [56]. In our case, the higher content of intramolecular Υ trimers of fish collagen may be correlated with the highest viscosity and lowest relaxation % of all collagens from Table 1. Thus, due to their different structure, it can be seen that the fish nanofibers presented the smallest modification of the FTIR spectra upon gamma irradiation while the bovine gelatin nanofibers showed the highest variation. These results correlate well with the EPR results, which emphasized the formation of very short life ^●^OH radicals upon irradiation only for bovine and donkey collagen nanofibers. Only minor shifts in FTIR bands were observed after irradiation, indicating the preservation of the secondary structure. Nevertheless, a slight increase in band intensity may be observed for the samples irradiated at 10 and 25 kGy, which may be due to cross-linking, as it is known that at low gamma doses the bond scission and cross-linking, which follow the ionization processes, are dominated by cross-links.

**Figure 7:**
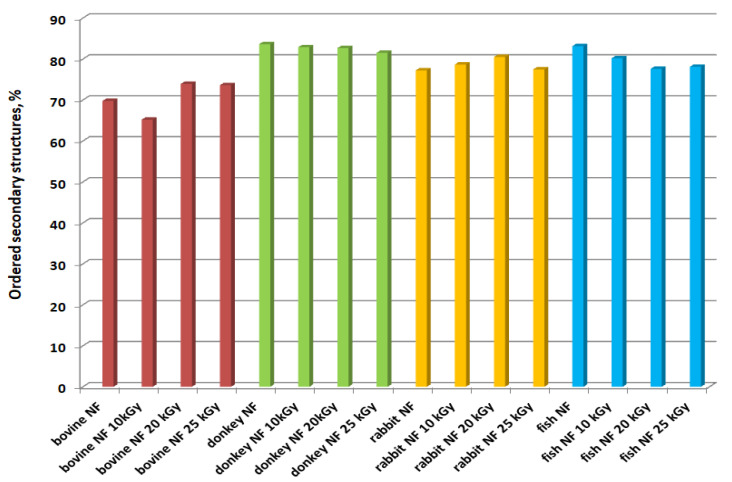
The ratio of ordered secondary structures of gelatin nanofibers of different origins and at different gamma radiation doses, determined by the deconvolution of the amide III band.

In order to confirm the main conclusions of ATR-FTIR analyses, deconvolution of the amide III band for gelatin nanofibers was performed. In Figure 7 and in SI (Figure S3 and Table S1), it can be seen that the treatment with gamma radiation of gelatin nanofibers of different origins did not have a significant influence on the ordered secondary structure of collagen. The recombination effects at 20 and 25 kGy are dominant for bovine gelatin nanofibers as well as for rabbit gelatin nanofibers, with the highest effect at 20 kGy and with a constant trend at all gamma radiation doses. Donkey gelatin nanofibers’ secondary structures were mostly influenced through molecule scission at 25 kGy; meanwhile, the fish scale gelatin nanofibers constantly lost the ordered structures under the influence of gamma radiation at all doses.

## 3. Conclusions

The influence of gamma irradiation doses (10, 20, and 25 kGy) on gelatin nanofibers fabricated from different-origin gelatins was evaluated compared to gelatins through microbial load measurements and spectral (EPR, ATR-FTIR) and light emission methods (chemiluminescence).

The efficiency of gamma irradiation doses on the total number of bacteria (TAMC), the total number of yeasts and filamentous fungi (TYMC), and the presence of *Staphylococcus aureus*, *Escherichia coli*, and *Candida albicans* strains revealed that all gamma irradiation doses were effective.

The microbial load was acceptable after gamma irradiation with a dose of 10 kGy according to requirements for pharmaceutical or topical products with minimum modifications of gelatin nanofiber structures. Donkey gelatin and rabbit gelatin showed the lowest stability at a temperature up to 200 °C; meanwhile, the gelatin nanofiber’s chemiluminescent signals were the most intense at a temperature up to 250 °C, suggesting oxidative processes that can occur in correlation with free radicals formed under gamma irradiation. Accordingly, the EPR signal was higher for bovine gelatin nanofibers and lower for donkey gelatin nanofiber, allowing us to identify the presence of HO^●^ radicals compared to gelatins and other nanofibers without the EPR signal. FTIR spectroscopy offered insight into the different primary and secondary structures of various marine and terrestrial mammalian collagen origins, justifying its particular behavior under gamma irradiation. Also, the FTIR spectra and amide III deconvolution of gelatin nanofibers confirmed the conservation of protein secondary structures (α-helix, β-sheet) through the slight shift in FTIR bands after gamma irradiation in correlation with EPR and chemiluminescence measurements.

## 4. Materials and Methods

### 4.1. Materials

Gelatins were prepared using bovine hide, donkey hide [P1], rabbit skin [P2], and fish scales [P3] at the Leather Research Department of ICPI (Bucharest, Romania). Bovine hide, donkey hide, and rabbit skin were processed through separation of the dermal layer from the hair and the hypodermal layer through chemical and mechanical treatments specific for leather manufacturing, followed by shredding the pelts and heating in a water bath at 90 °C for 4–12 h for gelatin extraction [34,47,54]. Fish scale gelatin was prepared by washing carp fish scales with 5% *w*/*v* NaCl and 4% *w*/*v* NaOH to release soluble proteins and then degreasing them with n-butanol. In order to solubilize the minerals, the fish scales were then subjected to a 16 h treatment with ethylene diamino tetraacetic acid (EDTA) and a 3 h treatment with acetic acid 99% *v*/*v*. Finally, chopped fish scales were heated at 60 °C for 12 h in distilled water in order to extract fish scale gelatin. Drying fish scale gelatin at 60 °C resulted in solid gelatin.

The reagents used to conduct tests were of analytical grade, suitable for microbiological use, as follows: water; tryptic soy broth culture medium (TSB); tryptone soy agar culture medium (TSA); enumeration agar (EA); culture medium of nutrient broth (NB); soybean casein digest lecithin polysorbate 80 medium (SCDLP); sabouraud dextrose agar medium (SDA); microorganism strains of *Staphylococcus aureus* ATCC 6538, *Escherichia coli* ATCC 10,536, and *Candida albicans* ATCC 10,231 (Mediclim, Otopeni, Romania).

### 4.2. Nanofibers (NF) Preparation

The gelatin dispersions were prepared by dissolving 12 g of solid gelatin in 100 mL of acetic acid of 90% *v*/*v* concentration. For nanofiber preparation, 10 mL of each gelatin solution was loaded in a 20 mL Teflon syringe equipped with a G21 needle in a uniaxial electrospinning device (TL-Pro-BM, Tong Li Tech Co., Ltd., Shenzhen, China). The nanofibers were obtained at a temperature of 29.6 °C and a relative humidity of 35% ± 5% using the optimized electrospinning parameters presented in Table 4.

### 4.3. Gelatin Nanofibers and Gelatin Treatment through Gamma Irradiation

A gamma irradiator M-38 GAMMATOR (Washington, DC, USA) was utilized to perform gamma irradiation using a ^137^Cs source in the air at room temperature. Parallel samples of gelatins and gelatin nanofibers were gamma irradiated with doses of 10, 20, and 25 kGy. Half of the irradiated samples of gelatins and nanofibers were immediately mixed with 5,5-dimethyl-1-pyrroline N-oxide (DMPO) and subjected to electron paramagnetic resonance analyses.

### 4.4. Characterisation Methods

#### 4.4.1. Physicochemical and Spectroscopic Characterization of Gelatins and Gelatin Nanofibers

Characteristics, such as volatile matter [60], total ash content [61], electrical conductivity [62], and pH of 10% *w*/*v* solution [63] were determined for all gelatins. The texture characteristics of gelatins were measured using TEX’AN TOUCH 50 N texture analyzer (LAMY RHEOLOGY, Champagne au Mont d’Or, France), the viscosity was measured with DV2T™ Viscometer Brookfield (Middleborough, MA, USA), and the conductivity was measured with potentiometer Orion Star™ A211 Benchtop.

SEM images were acquired using FEI Inspect S50 equipment—Scanning Electron Microscope. In order to avoid the charging effect, all samples were covered with a thin gold layer using a Cressington 108 auto sputter coater equipped with a Cressington mtm 20 thickness controller. The secondary electron images were obtained at a 10 mm distance using 5 kV acceleration voltage and magnification from 1000× *g* up to 10,000× *g*. The nanofiber’s average thickness was calculated as the mean diameter of a minimum of 50 nanofiber measurements, processed using ImageJ software version no. 1. 54d.

Chemiluminescence spectroscopy was performed in order to identify the nanofibers’ reaction to gamma irradiation and the ability of ordered structures of nanofibers to retain the active radical species generated by gamma irradiation. Non-isothermal emission intensity dependence on increasing temperature was recorded using the LUMIPOL 3 unit (SAS, Bratislava, Slovakia) chemiluminescence (CL) spectrometer. An aluminum cap containing 5 mg of nanofiber samples was electrically heated in a micro-oven set at an optimal heating rate of 5 °C min^−1^ starting from room temperature and ending at 250 °C ± 0.5 °C. The chemiluminescence values were normalized according to the nanofibers’ masses for reliable comparison. Similar analyses were performed for gelatins of nanofiber origin.

The electron paramagnetic resonance (EPR) spectra of spin adducts were recorded on a JEOL FA 100 spectrometer (Tokyo, Japan) equipped with a cylindrical TE011 resonator, with frequency modulation of 100 kHz, microwave power of 0.998 mW, sweep time of 60 s, modulation amplitude of 1 G, time constant of 0.1 s, modulation width of 1 G, and a magnetic field scan range of 100 G. The hyperfine coupling constants of the spin adducts were obtained as a result of the simulation of the experimental spectra performed using the Winsim software version 0.96 (Bethesda, MD, USA) [64,65].

A Bruker VERTEX 70 spectrometer (Bruker, Ettlingen, Germany) with 4 cm^−1^ resolution was used to record ATR–FTIR spectra. A total of 64 scans were performed in the 900–4000 cm^−1^ wavenumber range in order to obtain the background spectrum and sample spectra for non-irradiated and irradiated gelatins and gelatin nanofibers. Spectra were processed with the help of OPUS 7.5 software (Bruker, Ettlingen, Germany).

#### 4.4.2. Determination of Microbial Contamination

The microbial contamination of different-origin collagen nanofibers and gelatins was determined in agreement with the provisions of the European Pharmacopoeia Edition 10/2020.

The efficacy of gelatin and gelatin nanofibers preservation consists in placing the sample in an inoculum of appropriate microorganisms, storing this inoculated preparation at a preset temperature, and counting the organisms in the sample at a specified interval of time.

The preservation of the sample is considered adequate if in the conditions of the test, there is a significant reduction or no increase of microorganisms and the number of microorganisms complies with the protection intended for pharmaceutical products.

The method consists of interacting the gelatin nanofibers or gelatins with a concentration determined by the inoculum in order to establish their ability to reduce the initial concentration achieved by taking bacteria or fungi from the preserved stock. A plate containing EA was striped and incubated at 37 °C ± 20 °C for 24 h to 48 h, and then 20 mL of TSB was placed into a 100 mL Erlenmeyer flask. The initial cell concentration was previously established by decimal dilutions (10^5^), and at the last dilution, 100 µL was taken and spread onto nutrient agar for each strain. Colonies were counted from plates after 24 h of incubation and were then kept as references to determine cell growth in the control and test samples. Thus, plates with cell densities comparable to that of dilution 10^5^ were considered to have similar CFU values (1.2 × 10^5^ CFU/g for *Staphylococcus aureus*, 1 × 10^5^ CFU/g for *Escherichia coli,* and 2.5 × 10^4^ CFU/g for *Candida albicans*). Afterwards, 1.0 ± 0.1 mL of the inoculum was pipetted onto several points over each test sample. The samples were then placed in vials, which were shaken, immediately adding 20 mL of SCDLP medium. The vials containing the test material were placed in an incubator at 37 ± 1 ℃ for 18–24 h, then 1 mL of the inoculum was taken from the bacterial suspension in the sample, placed in a test tube incorporating 9.0 mL ± 0.1 mL of NB, and shaken well. The next step was to add 1 mL of this solution to a different test tube containing 9.0 mL ± 0.1 mL of medium and to shake it well. These operations were successively repeated 10 times in order to prepare a series of dilutions. Subsequently, 1 mL of each dilution was pipetted into two Petri dishes, while 15 mL of TSA was heated to 45 °C ± 1 °C in a water bath and added to the Petri dishes to number the colony-forming cell units. The number of colonies on the Petri dishes of the dilution series, on which 30 CFU/g to 300 CFU/g had appeared, was counted briefly after incubation.

The results represent the total number of bacteria (TAMC) constituting the average CFU determined on the agar medium with casein and soy hydrolyzate and the total number of yeasts and filamentous fungi (TYMC), namely the average CFU determined on the Sabouraud agar medium with chloramphenicol.

### 4.5. Statistical Data

The analyses’ results represent the arithmetic mean ± standard deviation (SD) of the mean value of at least 3 samples, and the significance of the differences recorded between the samples compared with the control group was determined by conducting one-way ANOVA in Microsoft Excel. Values of the *p* coefficient (probability) < 0.05 show that there are statistically significant differences between the means of nanofiber samples and the control sample.

## 5. Patents

**P1.**  Rapa, M.; Gaidau, C.; Matei, E.; Berechet, M.D.; Pantilimon, M.C.; Predescu, A.M.; Predescu, C. Composition of Nanowires Based on Collagen Using Rabbit Glue and Antimicrobial Agents and Process for Obtaining Them. RO 133873A0. Available online: http://pub.osim.ro/publication-server/pdf-document?PN=RO133873%20RO%20133873&iDocId=12868&iepatch=.pdf (accessed on 1 February 2024).
**P2.**  Rapa, M.; Gaidau, C.C.; Matei, E.; Stanca, M.; Berechet, M.D. Nanofibers from Donkey Hide Collagen and Process for Obtaining Therefore. OSIM A00770 from 28.11.2022 and Published in 2023. pg. 23. Available online: https://www.osim.ro/images/Publicatii/Inventii/2023/inv_03_2023.pdf (accessed on 1 February 2024).
**P3.**  Gaidau, C.; Rapa, M.; Predescu, C.; Stanca, M.; Alexe, C.A. Nanofibers from Fish Scale Collagen and Process to Obtaining Therefore, OSIM A00782 from 29.11.2022, Published in 2023. pg. 23. Available online: https://www.osim.ro/images/Publicatii/Inventii/2023/inv_03_2023.pdf (accessed on 1 February 2024).

## Figures and Tables

**Table 1:** The characteristics of gelatins for nanofiber preparation.

Characteristics	Gelatin Extracted From			
	Bovine Hide	Donkey Hide	Rabbit Skin	Fish Scales
Dry substance, %	7.69 ± 0.35	11.80 ± 0.35	7.30 ± 0.35	4.82 ± 0.35
Total ash *, %	7.67 ± 0.24	nd	0.68 ± 0.24	nd
pH (1:10), pH units	6.60 ± 0.10	6.70 ± 0.10	6.60 ± 0.10	4.91 ± 0.10
Bloom test, g	215.30	321.00	657.40	362.10
Relaxation, %	38.30	21.50	51.54	14.30
Viscosity, CP **	1.5	2.75	6.00	5.75
Shear stress, dyne/cm^2^	2.79	5.12	11.16	10.70
Conductivity, μS/cm	419 ± 0.10	53 ± 0.10	32 ± 0.10	442 ± 0.10

* values reported at dry substance; ** values measured at 60 °C.

**Table 2:** The efficacy of gamma irradiation for the preservation of gelatin nanofibers and gelatins against microbial contamination.

Samples	TAMC, CFU/g	TYMC, CFU/g	*Staphylococcus aureus*	*Escherichia coli*	*Candida albicans*
Nanofibers from bovine hide gelatin	1060 ± 11.846	966.6 ± 29.143	Positive	Positive	Positive
Nanofibers from bovine hide gelatin, irradiated at 10 kGy	2.33 ± 0.577	0	Negative	Negative	Negative
Nanofibers from bovine hide gelatin, irradiated at 20 kGy	0	0	Negative	Negative	Negative
Nanofibers from bovine hide gelatin, irradiated at 25 kGy	0	0	Negative	Negative	Negative
Bovine hide gelatin	0	0	Negative	Negative	Negative
Bovine hide gelatin, irradiated at 10 kGy	0	0	Negative	Negative	Negative
Bovine hide gelatin, irradiated at 20 kGy	0	0	Negative	Negative	Negative
Bovine hide gelatin, irradiated at 25 kGy	0	0	Negative	Negative	Negative
Nanofibers from donkey hide gelatin	1146 ± 4.509	986.6 ± 12.055	Positive	Positive	Positive
Nanofibers from donkey hide gelatin, irradiated at 10 kGy	0	0	Negative	Negative	Negative
Nanofibers from donkey hide gelatin, irradiated at 20 kGy	0	0	Negative	Negative	Negative
Nanofibers from donkey hide gelatin, irradiated at 25 kGy	0	0	Negative	Negative	Negative
Donkey hide gelatin	0	0	Negative	Negative	Negative
Donkey hide gelatin, irradiated at 10 kGy	0	0	Negative	Negative	Negative
Donkey hide gelatin, irradiated at 20 kGy	0	0	Negative	Negative	Negative
Donkey hide gelatin, irradiated at 25 kGy	0	0	Negative	Negative	Negative
Nanofibers from rabbit skin gelatin	0	0	Positive	Positive	Positive
Nanofibers from rabbit skin gelatin, irradiated at 10 kGy	0	0	Negative	Negative	Negative
Nanofibers from rabbit skin gelatin, irradiated at 20 kGy	0	0	Negative	Negative	Negative
Nanofibers from rabbit skin gelatin, irradiated at 25 kGy	0	0	Negative	Negative	Negative
Rabbit skin gelatin	0	0	Negative	Negative	Negative
Rabbit skin gelatin, irradiated at 10 kGy	0	0	Negative	Negative	Negative
Rabbit skin gelatin, irradiated at 20 kGy	0	0	Negative	Negative	Negative
Rabbit skin gelatin, irradiated at 25 kGy	0	0	Negative	Negative	Negative
Nanofibers from fish scale gelatin	86.00 ± 6.00	70.30 ± 1.528	Negative	Negative	Negative
Nanofibers from fish scale gelatin, irradiated at 10 kGy	0	0	Negative	Negative	Negative
Nanofibers from fish scale gelatin, irradiated at 20 kGy	0	0	Negative	Negative	Negative
Nanofibers from fish scale gelatin, irradiated at 25 kGy	0	0	Negative	Negative	Negative
Fish scale gelatin	0	0	Negative	Negative	Negative
Fish scale gelatin, irradiated at 10 kGy	0	0	Negative	Negative	Negative
Fish scale gelatin, irradiated at 20 kGy	0	0	Negative	Negative	Negative
Fish scale gelatin, irradiated at 25 kGy	0	0	Negative	Negative	Negative

**Table 3:** Main FTIR band positions (cm^−1^) of gelatin nanofibers and gelatins.

Material/ FTIR Bands	Nanofibers				Gelatins			
	Bovine	Donkey	Rabbit	Fish	Bovine	Donkey	Rabbit	Fish
Amide A	3285	3285	3287	3289	3283	3289	3279	3285
Amide B	2937	2940	2921	2937	2921	2936	2919	2939
Amide I	1640	1637	1640	1638	1638	1630	1620	1633
Amide II	1540	1538	1540	1538	1536	1543	1541	1532
Amide III	1242	1242	1243	1242	1241	1237	1241	1241

**Table 4:** Electrospinning parameters used for the preparation of gelatin nanofibers.

Parameters	Bovine Gelatin NF	Donkey Gelatin NF	Rabbit Gelatin NF	Fish Scale Gelatin NF
Flow rate, mL/h	1	0.8	0.7	2
Voltage supply, kV	23.20	22.59	24.35	23.24
Collector distance, mm	130	130	90	130

## Data Availability

The data presented in this study are openly available in the article.

## Supplementary Materials

The following supporting information can be downloaded at: https://www.mdpi.com/article/10.3390/gels10040226/s1, Figure S1: Chemiluminescence intensity at 200–250 °C for bovine collagen nanofibers (a) and gelatin (b); donkey collagen nanofibers (c) and gelatin (d); rabbit collagen nanofibers (e) and gelatin (f); fish scale collagen nanofibers (g) and gelatin (h); Figure S2: (a) Non-normalized spectra for bovine, rabbit, donkey, and fish gelatin nanofibers; (b) non-normalized spectra for bovine, rabbit, donkey, and fish gelatin nanofibers recorded after 30 min; Figure S3: Deconvolution of amide III band of rabbit gelatine nanofibers irradiated at 20 kGy; Table S1: Deconvolution of amide III with ratios of secondary structures.
