# Supplementary material for: The Influence of Gamma Radiation on Different Gelatin Nanofibers and Gelatins
Source: Gels. 2024 Mar 26;10(4):226. doi: 10.3390/gels10040226 (PMC11049530; doi:10.3390/gels10040226)
Supplement: Supplementary file 1 [file gels-10-00226-s001.zip › gels-2918716-supplementary.pdf]

The Influence of Gamma Radiation on Different Gelatin Nanofibers and Gelatins

Carmen Gaidau[1], Maria Râpă[2], Gabriela Ionita[3], Ioana Rodica Stanculescu[4,5], Traian Zaharescu[6], Roxana-Rodica Constantinescu[1], Andrada Lazea-Stoyanova[7] and Maria Stanca[1*]

2.4. Chemiluminescence Spectroscopy

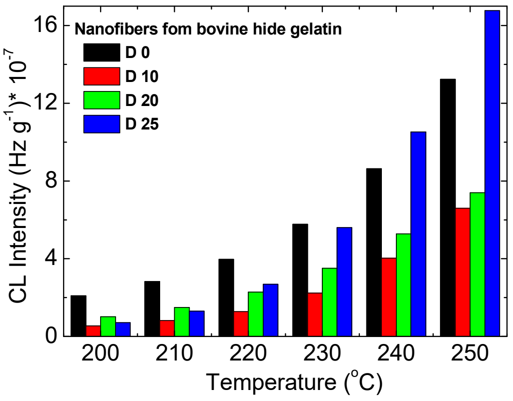

a)

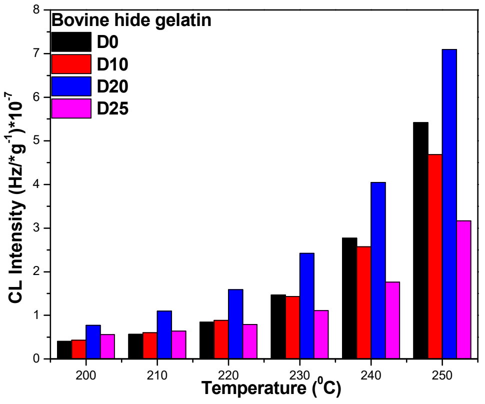

b)

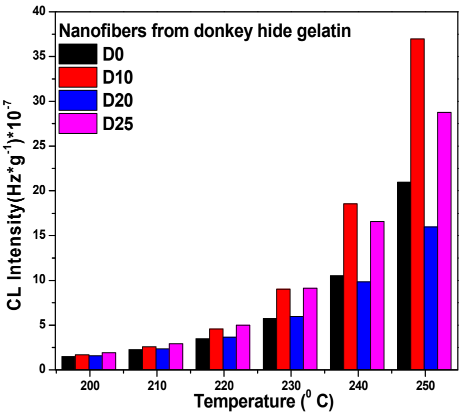

c)

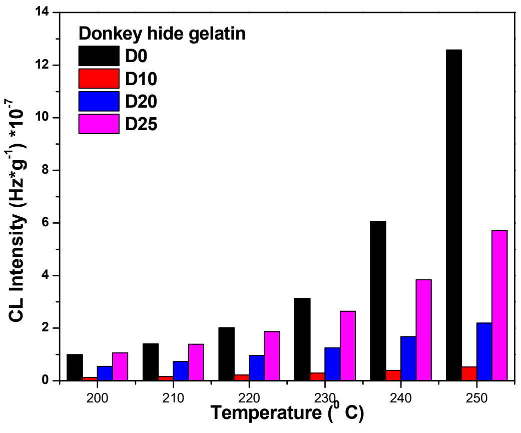

d)

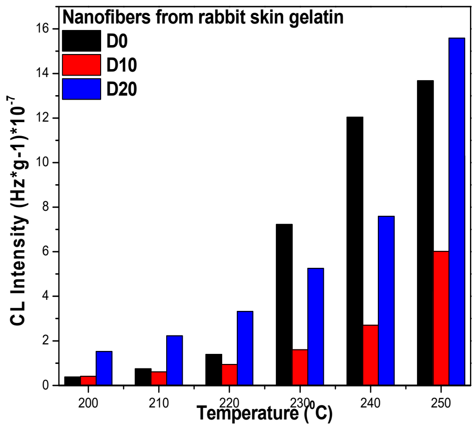

e)

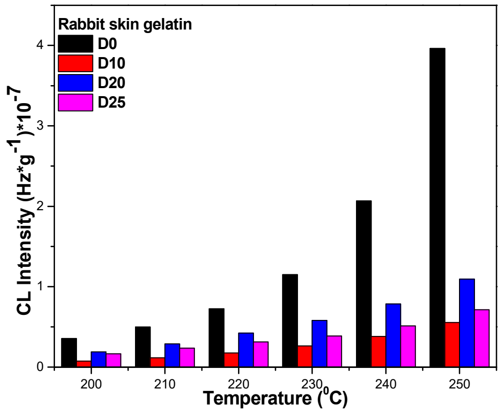

f)

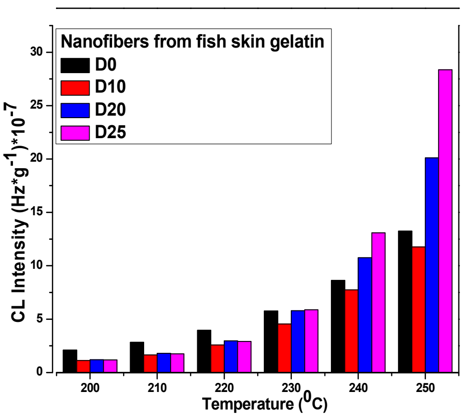

g)

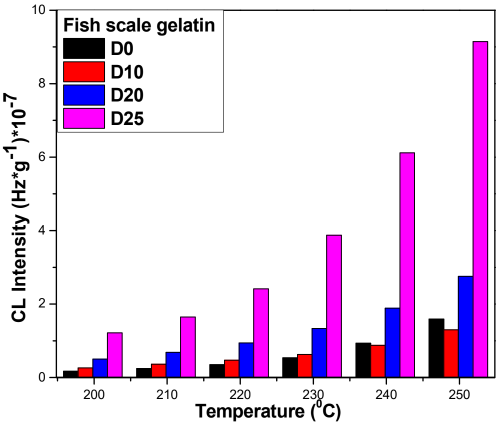

h)

Figure S1. Chemiluminescence intensity at 200-250^{0}C for bovine gelatin nanofibers (a) and bovine gelatin (b); donkey gelatin nanofibers (c) and donkey gelatin (d); rabbit gelatin nanofibers (e) and rabbit gelatin (f); fish scale gelatin nanofibers (g) and fish scales gelatin (h).

2.5. EPR Spectroscopy

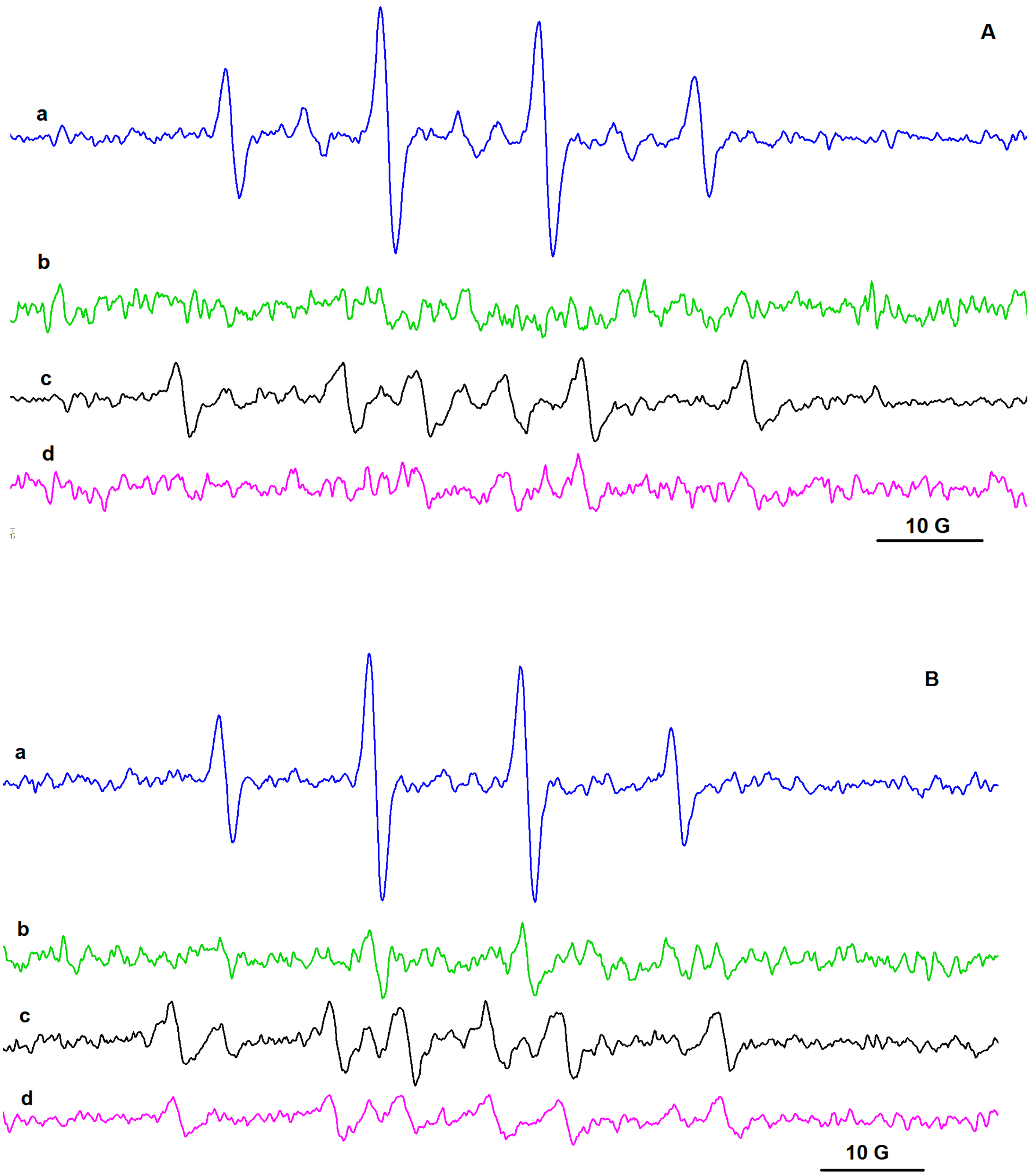

Figure S2. A) Non-normalized EPR spectra of DMPO adducts from bovine (a), rabbit (b), donkey (c) and fish gelatin (d) nanofibers samples; B) Non-normalized EPR spectra of DMPO adducts from bovine (a), rabbit (b), donkey (c) and fish gelatin (d) nanofibers samples recorded after 30 minutes.

2.6. ATR-FTIR Spectroscopy

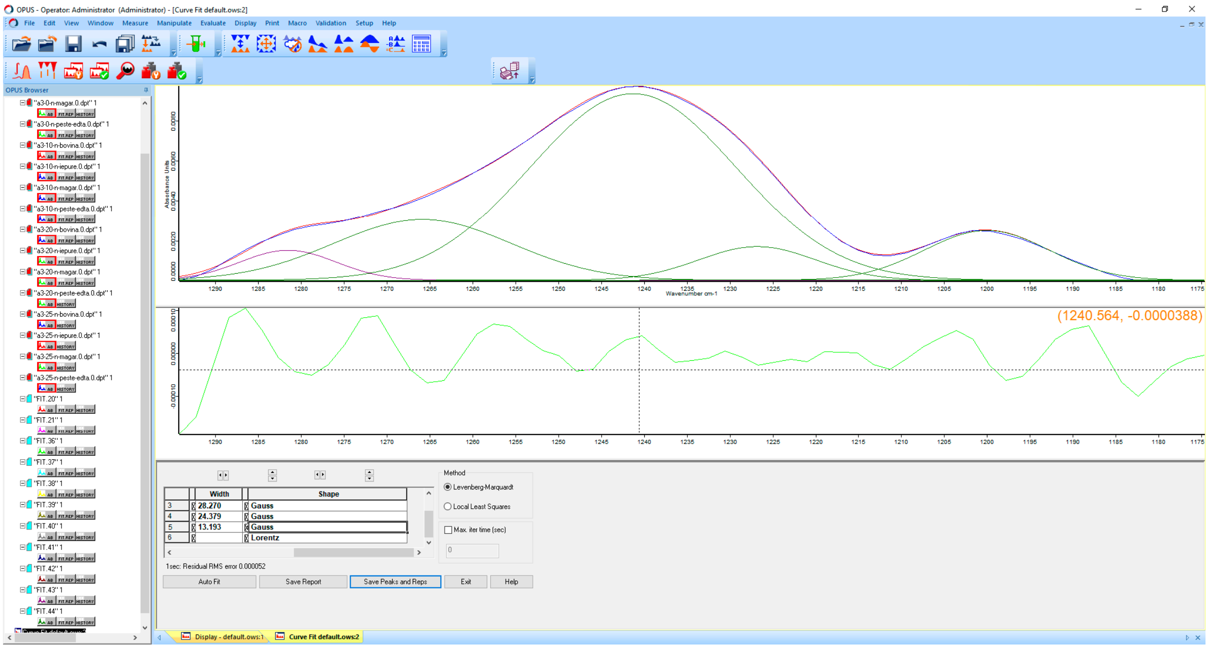

Figure S3 Deconvolution of Amide III band of rabbit gelatine nanofibers irradiated at 20 kGy

Table S1. S1 Deconvolution of Amide III with ratios of secondary structures

Sample	Deconvolution of Amide III with ratios of secondary structures, %				
	1200 cm^{-1} side amino acids	1226 cm^{-1} β-sheets	1240 cm^{-1} α-helix	1260 cm^{-1} random coil	1281 cm^{-1} β-turns
Nanofibers from bovine hide gelatin	7.15	8.84	48.31	30.22	5.46
Nanofibers from bovine hide gelatin, irradiated at 10 kGy	7.08	8.29	43.44	34.80	6.36
Nanofibers from bovine hide gelatin, irradiated at 20 kGy	9.28	8.10	51.41	26.06	5.13
Nanofibers from bovine hide gelatin, irradiated at 25 kGy	8.97	7.45	51.81	26.37	5.37
Nanofibers from rabbit skin gelatin	8.99	7.09	55.42	22.76	5.72
Nanofibers from rabbit skin gelatin, irradiated at 10 kGy	8.17	11.12	53.78	21.33	5.58
Nanofibers from rabbit skin gelatin, irradiated at 20 kGy	9.79	3.50	62.28	19.48	4.93
Nanofibers from rabbit skin gelatin, irradiated at 25 kGy	9.81	6.83	55.29	22.52	5.53
Nanofibers from donkey hide gelatin	10.05	5.75	63.28	16.36	4.53
Nanofibers from donkey hide gelatin, irradiated at 10 kGy	9.83	5.59	62.55	17.11	4.90

Nanofibers from donkey hide gelatin, irradiated at 20 kGy	9.95	6.30	61.79	17.33	4.61
Nanofibers from donkey hide gelatin, irradiated at 25 kGy	10.29	5.31	61.54	18.47	4.37
Nanofibers from fish scales gelatin	7.88	2.33	69.26	16.85	3.66
Nanofibers from fish scales gelatin, irradiated at 10 kGy	7.89	5.29	62.93	19.82	4.06
Nanofibers from fish scales gelatin, irradiated at 20 kGy	7.72	4.46	61.33	22.39	4.07
Nanofibers from fish scales gelatin, irradiated at 25 kGy	7.76	4.69	61.39	21.88	4.25
